# Perioperative Management of Antithrombotic Therapy in Patients Who Undergo Dental Procedures: A Systematic Review of the Literature and Network Meta-Analysis

**DOI:** 10.3390/ijerph20075293

**Published:** 2023-03-28

**Authors:** Andrea Boccatonda, Alessio Frisone, Felice Lorusso, Calogero Bugea, Maristella Di Carmine, Cosima Schiavone, Giulio Cocco, Damiano D’Ardes, Antonio Scarano, Maria Teresa Guagnano

**Affiliations:** 1 Internal Medicine, Bentivoglio Hospital, AUSL Bologna, 40010 Bentivoglio, Italy; 2 Department of Innovative Technologies in Medicine & Dentistry, University of Chieti-Pescara, Via Dei Vestini 31, 66100 Chieti, Italy; 3 Department of Medicine and Science of Aging, “G. d’Annunzio” University, 66100 Chieti, Italy; 4 Department of Oral Implantology, Dental Research Division, College Ingà, UNINGÁ, Cachoeiro de Itapemirim 29312, ES, Brazil

**Keywords:** tooth extraction, oral surgery, haemostasis, oral haemorrhage, anticoagulants, antiplatelet therapy, antithrombotic therapy, atrial fibrillation and oral surgery

## Abstract

Background: The surgical dental treatment of subjects admitted for anticoagulants therapy represents a consistent risk for peri-operative bleeding. The aim of the present study was to investigate the clinical findings of dental surgery operative management of the patients under anticoagulants drugs protocol. Methods: The literature screening was performed using Pubmed/Medline, EMBASE and Cochrane library, considering only randomized clinical trials (RCTs) papers. No limitations about the publication’s period, follow-up time or clinical parameters were considered. Results: A total of eight RCTs were included for the qualitative synthesis. No thromboembolic complications were reported in any studies. Several bleeding episodes associated with anticoagulant drugs in dental surgery were mild and generally happened on the first day after the treatment. Conclusions: The use of local haemostatic measures is generally effective for bleeding control with no further pharmacological drug management or suspension.

## 1. Introduction

When a patient treated with oral anticoagulants must undergo an elective procedure, the risk of bleeding must be weighed against the risk of thrombosis associated with the interruption of anticoagulant medication. Dental procedures can be divided into those at high risk of bleeding and those with low risk of bleeding. Low-risk procedures such as scaling and/or root planing, restorative treatment, non-surgical endodontic treatment, simple extractions or minor surgery usually do not need any change in the antithrombotic regimen, as the risk of thrombosis far outweighs the risk of bleeding. Surgical extractions, multiple extractions, complex oral surgery, or head and neck cancer surgery are related to a high risk of bleeding, thus requiring a more complex decision on antithrombotic treatment change to prevent uncontrolled bleeding [1,2]. Moreover, some patients are characterized by a complex medical history, such as having liver disease, biliary tract obstruction, malabsorption, infectious diseases, genetic coagulation disorders, chronic inflammatory diseases, chronic renal disease, leukaemia, or other types of cancer that can influence the choice of antithrombotic prescription. Moreover, patients who have undergone chemotherapy or radiation therapy or have been exposed to large amounts of radiation are at higher risk of bleeding than healthy subjects [3]. The aim of this systematic review and network meta-analysis is to provide practical information on the management of anticoagulant and antiplatelet therapy in patients undergoing dental procedures.

## 2. Materials and Methods

### 2.1. Database Search Strategy

The present review has been registered on PROSPERO electronic database (n. 399590). The article database search was conducted in accordance to the Standards for Reporting Qualitative Research (SRQR) and the PRISMA guidelines [4,5,6]. The keywords search was based on the search strategy detailed in Table 1. The database screening was assessed and updated to 29 October 2022.

### 2.2. Inclusion and Exclusion Criteria

The first level, abstract/title screening, considered the manuscripts according to the following inclusion criteria: randomized clinical trials, or prospective and retrospective studies. The exclusion criteria for the present study were systematic reviews, editorial articles, papers written in a language other than English, case reports/series, and in vitro studies. The manuscript included were considered for full-text evaluation. 

### 2.3. Paper Selection Procedure

The eligibility assessment was performed independently by two expert reviewers (F.L. and A.S.). Articles in the English language that followed the inclusion criteria were included and the full text was obtained. Duplicates and excluded articles were categorized, recording the reasons for exclusion.

### 2.4. Study Assessment

The research data were carefully elaborated through a special designed Excel database (Microsoft, Redmond, WA, USA) according to the following categories: authors, journal, years of publication, study design, anticoagulant protocol, subjects (age, gender), haemostatic agents applied, dental treatment, sites, INR, complications, bleeding time-point, haemostasis time, and related thromboembolic events.

### 2.5. Risk of Bias Assessment

The risk of bias assessment was performed using the software Rev Man 5.5 (The Nordic Cochrane Centre, The Cochrane Collaboration, Copenhagen, 2014). The OHAT Guidelines for Risk of Bias Rating Tool for Human and Animal Studies was considered for the present analysis. The following criteria were applied: randomization sequence, allocation concealment, blinding participants, blinding outcomes, incomplete outcome data, selective reporting, and other biases. The risk of bias criteria was categorized according to the following categories: adequate, unclear, or inadequate. The articles selected were considered a low risk of bias, with a minimum ratio of 5/7 positive parameters. Otherwise, the articles were categorized at high risk. 

### 2.6. Comparative Meta-Analysis

A pragmatic computational model was adopted for the network meta-analysis concerning Owen et al.’s previously described method [7]. A network model concerned the relative risk of three different haemostatic agent approaches: gauze compression, gauze/tranexamic acid, and fibrin sponge being applied [8,9]. In another instance, a second network model was adopted concerning pharmacological therapy management: full coumarins (full-CU) dosage, reduced coumarins (low-CU) dosage, 2 days suspension (2-DSu), and low-weight heparin (Hep) [10,11,12].

## 3. Results

### 3.1. Articles Screening

**Figure 1:**
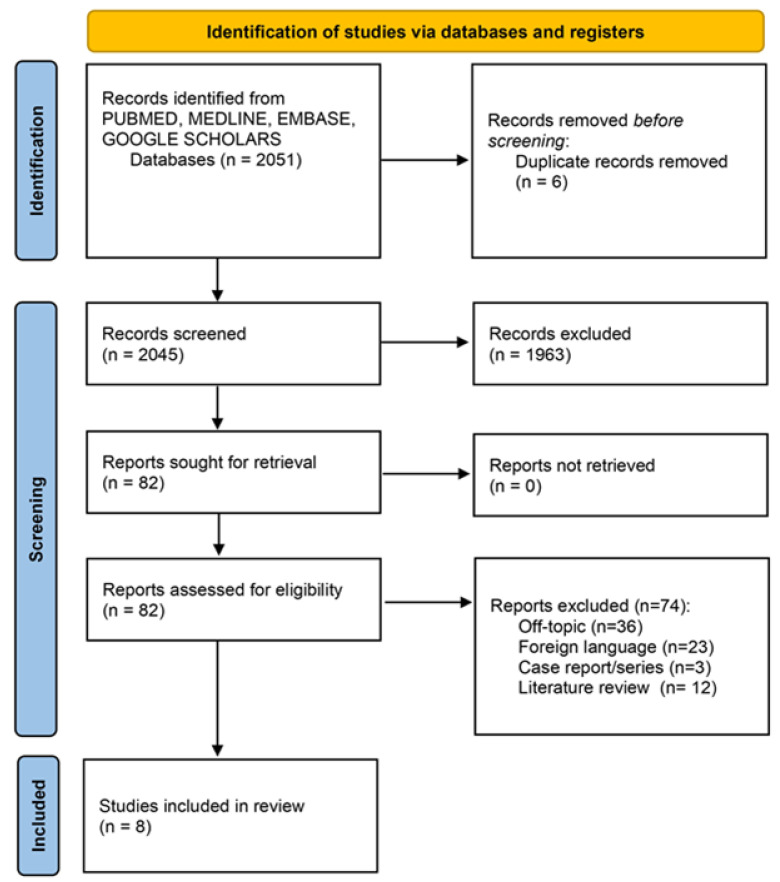
Summary of the screening process in accordance with the PRISMA guidelines [6].

The articles’ initial identification, eligibility and inclusion process was described in Figure 1. The scientific paper list included a total of 2051 manuscripts, and six duplicates have been removed. After the title and abstract evaluation, a total of 1963 manuscripts were excluded after the screening phase and 82 papers were included for the full-text evaluation. A total of 74 full-text papers was excluded: 36 off topic articles, 23 papers written in a non-English language, three case reports/series, and 12 literature reviews. A total of eight papers were included for qualitative synthesis and meta-analysis assessment.

### 3.2. General Characteristics

All studies included considered randomized clinical trial study designs [8,9,10,11,12,13,14,15], while one study included a double-blind procedure [8] and one study used a split mouth protocol [14]. In all cases, the pharmacological class of coumarins vitamin K antagonist full therapy (rivaroxaban, apixaban, or endoxaban excluded) was evaluated [8,9,10,11,12,13,14,15], in one case the coumarins administration was also reduced [11], in two studies a coumarins suspension of 2 days was performed before the surgery [12,15], and in one study a low-molecular-weight heparin administration was given [10]. The most common bleeding timepoint was detected in the immediate peri-operative period (<24 h) [8,9,10,11,12,13,14,15], and delayed bleeding was reported by four studies [10,11,13,15]. No other significance complications/thromboembolic events were reported in all cases [8,9,10,11,12,13,14,15] (Table 2). 

### 3.3. Risk of Bias Assessment Findings

**Figure 2:**
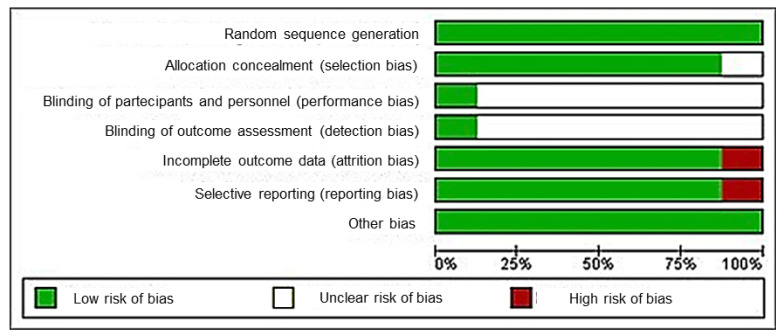
Risk of bias graph about each risk of bias item presented as percentages across all included studies (green dot: low risk of bias; red dot: high risk of bias).

**Figure 3:**
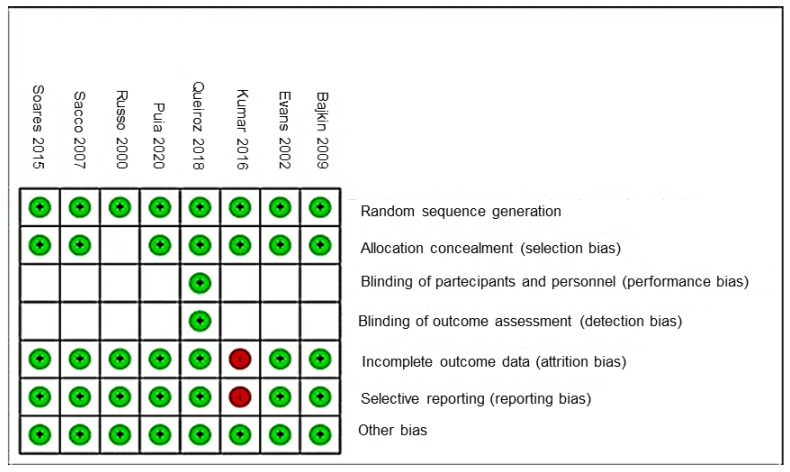
Risk of bias summary about each risk of bias item for each included study (green dot: low risk of bias; red dot: high risk of bias) [8,9,10,11,12,13,14,15].

The risk of bias findings are reported in Figure 2 and Figure 3. The most recurrent parameters were performance and detection biases, with a high percentage of uncleared risk (>75%), followed by the reporting and attrition biases. The study of Lourenço-Queiroz et al. [8] was the only randomized clinical trial (RCT) that reported the maximum score of 7/7 risk of bias assessment (Figure 3). According to previously described methods, the low-risk studies were included for further network meta-analysis approaches [8,9,10,11,12].

### 3.4. Network Meta-Analysis and Crossed Treatment Comparison

#### Anticoagulants Dosage

**Figure 4:**
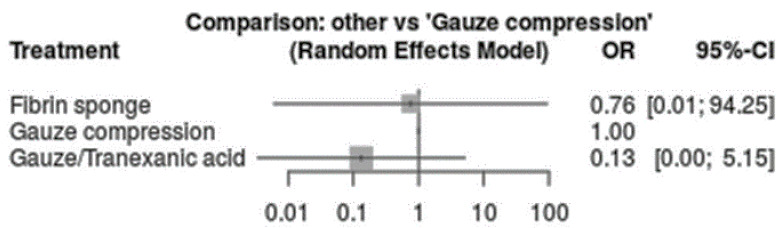
Intervention regarding the local haemostatic agents application compared to “gauze compression”.

**Figure 5:**
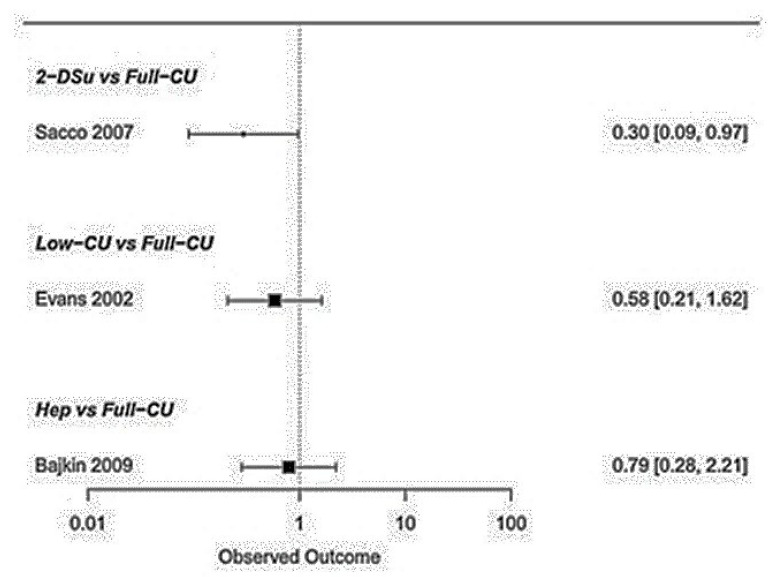
Intervention comparison regarding the different anticoagulants’ protocols [10,11,12].

**Figure 6:**
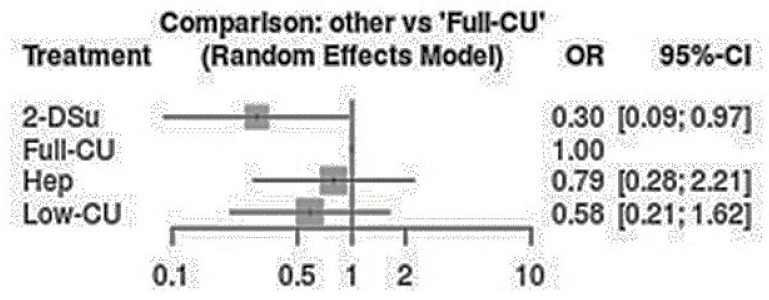
Intervention regarding the anticoagulant protocols compared to continuous full coumarins therapy.

Regarding the haemostatic agents, the 2 day cumarins suspension showed a significantly lower risk of bleeding compared to the continuous cumarins full therapy (*p* < 0.05) [OR: 0.30; 95%CI: 0.09–0.97]. Similar findings were detected between the low-dosage vs. continuous cumarins full therapy (*p* > 0.05) [OR: 0.58; 95%CI: 0.21–1.62] (Figure 4, Figure 5 and Figure 6). No significant differences were detected between low-molecular-weight heparin vs. continuous cumarins (*p* > 0.05) [OR: 0.79; 95%CI: 0.28–2.21].

## 4. Discussion

### 4.1. Perioperative Management of Antiplatelet Drugs

Current evidence and guidelines are against discontinuing antiplatelet therapy in subjects undergoing dental procedures. The present literature review evidenced non-homogeneous protocols mainly considering four different approaches: a non-discontinuing coumarins suspensions, a reduction of the coumarins dosage, a substitutive therapy with low-molecular-weight heparin, and finally a 2 day complete suspension of anti-coagulants [8,9,10,11,12,13,14,15]. Due to the emerging of novel classes of antithrombotic drugs, haemostatic agents, the theme can still be seen, with >100 scientific contributions in the last 5 years. Dis-homogeneous pharmacological protocols and drugs administrations have been proposed according to the severity of the bleeding alteration and the invasiveness of the surgical procedure. For this purpose, this could be considered a limit for the statistical analysis of the meta-data that could result in a weaker power of the calculation. On the other hand, a network meta-analysis on low risk-of-bias level studies is able to offer the higher as a possible level of evidence of the current scientific literature. In addition, the present investigation offers a novel approach, as no studies with network meta-analysis have yet been published in the literature. In the first instance, the first pre-operative International Normalized Ratio ration looks consistently dis-homogeneous when comparing between the included studies, but in all cases INR < 3.0 [8,9,10,11,12,13,14,15]. Post-operative bleeding was most commonly reported in the immediate post-operative period <1st day that was controlled in almost all clinical situations with local haemostatic/gauze compressions and additional sutures. A limited quantity of cases presented mild bleeding that required tranexamic acid mouthwash. Puia et al. considered the use of a bismuth subgallate (BS) plug, fibrin tissue adhesive (FTA) or microfibrillar collagen (MC) as effective devices for the haemostatic control of alveolar bleeding, with very limited post-operative events: 11 cases/240 subjects with no anticoagulants suspension [13]. In all studies included, no additional complications/thromboembolic events were reported [8,9,10,11,12,13,14,15]. Regarding the network meta-analysis outcome, a reduced quantity of randomized clinical trials (RCTs) at low risk of bias was detected by the screening process. On the other hand, the anticoagulant drug protocols, the 2 day cumarins suspension group, reported a lower clinical risk of bleeding compared to the full continuous cumarins therapy (*p* < 0.05). No differences were detected for heparin and low-dosage cumarins compared to the standard protocol. Another important finding was characterized by the local haemostasis agents’ administration. In fact, the combination of gauze and tranexamic acid was reported as the most effective for early bleeding administration (*p* < 0.05). Alveolar socket bone bleeding represents a remarkable clinical occurrence in patients with coagulation disorders and INR > 2.0. An alternative approach could be represented by using calcium sulphate, which several studies suggested in relation to very effective haemostasis and the contextual promotion of the in-site bone regeneration [16,17,18]. A previous review performed by Napenas et al. demonstrated no significant risk of postoperative bleeding in patients undergoing invasive dental procedures on either single or dual antiplatelet therapy [19]. Another work, performed on 14,981 patients in whom perioperative continuation was compared with the discontinuation of low-dose acetylsalicylic acid, demonstrated that 0.6% of patients with therapy suffered cardiovascular events [20]. Furthermore, a meta-analysis performed on 50,279 subjects demonstrated that acetylsalicylic acid discontinuation was related to a threefold increased risk of severe cardiovascular events, with an even more increased risk (OR 89.78) for patients with intracoronary stents [21]. Clopidogrel can be continued for dental surgical procedures [3], but it may also be discontinued seven days before surgery in patients at low thrombotic risk and resumed after surgery once haemostasis is achieved [22,23]. In cases of patients on dual antiplatelet therapy (DAPT), its discontinuation is related to a five- to ten-fold increased risk of myocardial infarction in patients with coronary stents, and the risk is inversely correlated with the timing of previous cardiac interventions [24]. Moreover, only mild bleeding has been detected in the setting of dental surgical procedures performed on patients on DAPT [19,25]. Bleeding can be stopped by using local haemostatic measures only [26,27]. A systematic Cochrane review demonstrated a positive effect of locally applied tranexamic acid (mostly 5% concentration, for 3–4 days) to prevent oral bleeding events in patients receiving oral anticoagulation (vitamin K antagonists or DOACs) undergoing dental surgery [28]. Therefore, the American Dental Association (ADA) suggests that there is no need to discontinue antiplatelet therapy before dental procedures in healthy subjects, and that local haemostatic measures are sufficient [29]. 

### 4.2. Perioperative Management of Heparin

Surgery can be performed 2–4 h after heparin discontinuation. Patients taking LMWH can undergo invasive dental procedures 12 h after drug discontinuation [3]. Local haemostatic measures should be used in the case of postoperative bleeding. LMWH therapy can be resumed after achieving haemostasis. 

### 4.3. Preoperative Management of Warfarin

There is evidence to continue warfarin therapy in patients undergoing minor dental procedures or other invasive dental procedures when INR values do not exceed 3.5. A previous meta-analysis did not demonstrate increased bleeding risk with continued warfarin therapy, when compared to treatment discontinuation or dose adjustment in patients undergoing single or multiple extractions [30]. Those data were supported by a subsequent systematic review by Weltman et al. [31], showing that patients with an INR within the therapeutic range can safely continue taking the regular dose of warfarin before dental extractions. In a systematic review, Siegal et al. argued that patients who received “bridging” therapy with heparin, compared with a group in which oral vitamin K antagonist therapy was continued, had a higher bleeding risk and a similar rate of thromboembolic events [32]. Douketis et al. performed a study on patients on warfarin therapy for atrial fibrillation and compared 891 bridged patients with 913 patients receiving a placebo before surgery; there was no significant difference in the rate of thromboembolic events between the groups, and bleeding occurred significantly more frequently in the bridging group [33]. A subsequent review by Young et al. confirmed that finding, thus showing that bridging was more often related to bleeding events (odds ratio [OR]: relevant bleeding 3.23; OR minor bleeding 1.52), and no differences in thromboembolic events were demonstrated [34]. Hiroshi et al. performed a cross-sectional study, evaluating data from 496 patients in whom warfarin had been continued for tooth extraction (INR ≤ 3, 7 days before intervention) in comparison with 2321 patients in whom vitamin K antagonist therapy was discontinued. Bleeding events were significantly more frequent in the group that had maintained anticoagulant therapy; age of <65 years, higher pretherapeutic INR, concomitant antiplatelet agents, and the presence of inflammation at the extraction site were related to the risk of more severe bleeding events [35]. In contrast, another meta-analysis did not demonstrate a significant difference in postoperative bleeding of 10.8% with continued oral anticoagulation versus 8.3% with discontinuation of anticoagulation for tooth extraction. Notably, local haemostatic measures were sufficient to reach bleeding control in almost all studies [36]. Otherwise, no clear evidence is present on major surgical procedures. In patients with liver disease or kidney disease or taking additional drugs (e.g., aspirin, antibiotics, nonsteroidal anti-inflammatory drugs [NSAIDs]), anticoagulant treatment must be planned individually.

### 4.4. Perioperative Management of Doacs

As regards the perioperative management of DOACs on dental surgery, there are fewer data in the literature, but the shorter half-life and the safety and efficacy characteristics of these drugs make them more manageable than vitamin K antagonists. As evidenced by a previous position paper, low bleeding risk surgery does not require DOAC interruption in subjects with normal renal function [37]. It is advisable that the procedure is performed at trough DOAC concentrations, i.e., 12 or 24 h after the last intake depending on twice-daily or once-daily dosing [37]. Therefore, procedures performed at peak plasma concentration should be avoided [38]. The latest EHRA guidelines confirmed that there is no indication to bridging with heparin [38]. In patients with comorbidities favouring the accumulation of the drug (kidney disease, advanced age, etc.), DOAC interruption 12–24 h before surgery can be considered [38]. If the procedure is considered high-risk, it is suggested to reintroduce the full dose of anticoagulant at 48 h, while the recovery should be at 24 h in case of low-risk intervention [38]. In the case of dental procedures with a higher risk of bleeding, it is suggested to delay the morning dose of once-daily agents (rivaroxaban, edoxaban) on the day of dental treatment, and skip one dose of twice-daily medications (apixaban, dabigatran) [37]. In subjects taking rivaroxaban or edoxaban in the evening, there is no need to modify their medication schedule before dental treatment [37]. If complete haemostasis has been achieved, DOAC can be resumed six to eight hours after the intervention [37]. Recent evidence in the literature reinforces the concept of not withholding DOAC therapy, especially in the case of interventions with low and medium risk of bleeding. Hanken et al. evaluated 52 dental surgical procedures (osteotomy, implantation) under Rivaroxaban (20 mg/days), with 285 procedures without anticoagulation. A significantly higher bleeding rate (11.5% versus 0.7%) was found in the DOAC group [39]. However, no difference has been demonstrated in other works [40,41]. A subsequent work performed on 12 patients receiving Rivaroxaban (discontinued 24 h before the procedure) who placed 57 implants showed no postoperative bleeding events [40]. A recent review did not demonstrate any differences in bleeding and thrombotic events in patients undergoing invasive dental procedures, while DOAC was either continued or discontinued for a short period [42]. No differences in terms of post-operative bleeding were found in a recent study comparing 31 patients on continued DOAC medication with 20 patients on continued vitamin K antagonization for tooth extraction. In particular, all bleeding events in the DOAC group occurred in patients in whom the intervention took place <4 h after the last dose of the drug [43]. Therefore, in the case of urgent surgery, it is advisable to delay surgery by at least 4 h (pharmacologically reasonable 12–24 h) after the last DOAC administration, because a substantial amount of the drug is eliminated within this period of time [44]. Bridging with heparin is currently not recommended with DOAC [45]. We report below the evidence divided for each individual drug. A review was performed on DOAC management in patients subjected to dental procedures with a low (e.g., local anaesthesia, simple extractions, supra-gingival scaling, single tooth extractions) to moderate (e.g., extractions of two to four teeth and a local periodontal surgery involving up to five teeth) risk of bleeding [46]. Data from that review demonstrated that the risk of bleeding was low regardless of whether or not the drug was discontinued before the procedure, and that haemostasis can be reached with local measures [46]. Another systematic review argued that, for most dental procedures, there is no need to discontinue anticoagulant drugs [47]. Otherwise, a multidisciplinary approach is suggested for more complex patients and/or high bleeding risk procedures.

## 5. Conclusions

The main evidence of the present investigation is that a drug holyday of anticoagulant or antiplatelet therapy for dental surgery seems to produce no increase of the clinical risk of bleeding or severe complications related to the procedures. Withdrawal or heparin bridging therapy were related to a greater risk of thrombotic events than bleeding events. Most bleeding events during antithrombotic therapy in dental surgery are mild and treatable with local haemostasis measures. In any case, it is always necessary to stratify the risk of the intervention and the type of patient. In the case of surgery with a high risk of bleeding or a patient with comorbidities favouring drug accumulation, it is advisable to perform a more precise assessment and prescribe a personalised therapy. Further studies are needed to better define the safety of DOACs in some specific patient settings, such as a patient with renal insufficiency. The advent of new and future anticoagulant drugs (e.g., anti factor XIa) could further reduce the risk of bleeding during dental procedures.

## Figures and Tables

**Table 1:** Keyword strategy for database search.

	Search Strategies
Keywords Search:	Advanced keywords search: (bone regeneration OR dental implants OR teeth extraction OR oral surgery) AND (anticoagulants OR warfarin OR direct oral anticoagulants OR DOAC OR rivaroxaban OR apixaban OR dabigatran) AND (bleeding AND complications)
Electronic Databases	*Pubmed/Medline, EMBASE, Google scholars*

The following PICO question was considered: P = Population/Patient/Problem—Subjects under antithrombotic/anticoagulants treatment; I = Intervention—Dental surgery procedure; C = Comparison—Drug holyday/suspension vs. no pharmacological administration variations; O = Outcome—Measurements of the bleeding complications and major events.

**Table 2:** Summary of the studies included for the qualitative synthesis [RCT: randomized clinical trial].

Authors	Journal	Year	Study Design	Blinding	Anticoagulant Protocol	Subjects	Gender	Age	Haemostatic Agents	Dental Treatment	Complication Site (s)	INR Mean	Post Operative Complications	Bleeding Timepoint	Time to Haemostasis	Thromboembolic Events
Puia et al. [13]	Ann Maxillofac Surg	2020	RCT	-	Vitamin K antagonist	240 subjects (80 per group); 267 extractions	94 male 146 female	60.5 ± 14.5 years	(1) bismuth subgallate (BS) plug (2) fibrin tissue adhesive (FTA) (3) microfibrillar collagen (MC)	Simple dental extractions	4 bleeding maxilla 7 bleeding mandible	2.62	BS: no complications FTA: 1 bleeding MC: 10	4 bleeding (day 1) 6 bleeding (day 2) 1 bleeding (day 3)	-	None
Lourenço Queiroz et al. [8]	Clin Oral Investig	2018	RCT	Double-blind	Vitamin K antagonist	40 subjects	6 male 14 female	45.5 ± 15.9 years	(1) saline gauze compression and suture (2) irrigation and compression with gauze/Tranexanic acid (TA) (250 mg/5 mL) and suture	Simple dental extractions	-	2.4 ± 0.7	(1) 20 bleeding (2) 13 bleeding	22 bleeding (day 1)	(1) 11.9 ± 2.5 (2) 5.9 ± 1.4	-
Kumar et al. [14]	J Clin Diagn Res	2016	RCT	Split mouth	Vitamin K antagonist	30 subjects	12 males 18 female	18–90 years	(1) chitosan based plug; (2) saline gauze compression	Simple dental extractions	-	<4	-	-	(1) 1.49 ± 0.39 (2) 4.6 ± 1.85	-
Soares et al. [9]	Oral Maxillofac Surg.	2015	RCT	-	Vitamin K antagonist	38 subjects 84 surgeries	56 male 28 female	51.1 ± 3.0 years	(1) gauze pad soaked 4.8% TA (2) fibrin sponge (3) gauze compression	Simple dental extractions		2.5 ± 0.1	(1) 1 bleeding (2) 2 bleeding (3) 1 bleeding	4 bleeding (day 1)	-	-
Bajkin et al. [10]	J Oral Maxillofac Surg.	2009	RCT	-	(1) vitamin K antagonist (2) bridging therapy with low-molecular-weight heparin	214 subjects [1: 109 patients; 2:105] 385 extractions	123 male 91 female	62.1± 11.4 years 59.6 ± 11 years	New sutures, compression,	Simple dental extractions	-	2.45 ± 0.54.	(1) 8 bleeding(2) 5 bleeding	9 bleeding (day 1) 7 bleeding (day 2)	-	None
Sacco et al. [11]	Oral Surg Oral Med Oral Pathol Oral Radiol Endod.	2007	RCT	-	(1) vitamin K antagonist full therapy (2) reduced anticoagulant therapy	131 subjects 511 extractions	29 male 36 female	(1) 64.0 ± 11.0 years (2) 61.5 ± 12.7 years	New sutures, clot removed, local haemostatic agents, and tranexamic acid mouthwashes.	Simple dental extractions, Third molars extractions	-	1.77 ± 0.26 2.89 ± 0.42	(1) 10 bleeding (2) 6 bleeding	12 bleeding (day 1) 4 bleeding (day 2)	-	None
Evans et al. [12]	Br J Oral Maxillofac Surg.	2002	RCT		(1) continuous vitamin K antagonist (2) anticoagulant stopped 2 days before surgery	109 subjects	(1) 36 male; 21 female (2) 37 male; 15 female	(1) 67.0 years (2) 66.0 years	New sutures, compression, antibiotic therapy, additional analgesia	Simple dental extractions	-	(1) 2.5(2) 2.6	(1) 7 bleeding (2) 15 bleeding	(1) 12 bleeding (day 1) (2) 7 bleeding (day 1)	-	-
Russo et al. [15]	Clin Appl Thromb Hemost	2000	RCT	-	Anticoagulant stopped 2 days before surgery	104 subjects 123 procedures	48 male 56 female	63.0 years old	New sutures, compression, tranexamic acid mouthwash, reduction of warfarin dosage	Simple dental extractions, gingival surgery, alveolar bone	-	2.95 ± 0.59	2 bleeding	1 bleeding (day 2) 1 bleeding (day 5)	-	None

## Data Availability

All experimental data to support the findings of this study are available upon request by contacting the corresponding author. The authors have annotated the entire data building process and empirical techniques presented in the paper.
